# Dr. Clay on the Value of Ox-gall

**Published:** 1842-09-03

**Authors:** 


### ANALECTA.

A writer in the Medical Times, Dr. Clay, of Manchester, has published an interesting communication on the value of ox-gall as a medicinal agent. Not having the journal before us, we extract a portion of the paper as re-published in the Medico-Chirurgical Review, trusting that some of our readers will subject the remedy to further trial.

So far as these experiments have progressed, the use of ox-gall in some diseases is of the most satisfactory character, presenting us with an excellent and peculiarly effective corrective for the many and various derangements of the alimentary canal, unlike many of our best medicines, inasmuch as in whatever cases it is given, if no benefit results, no harm is ever experienced from it. Its action on the system is not as a purgative, but as a mere solvent of the *material* contained within the intestinal canal, producing no excitement to propel, but by liquifying the mass, facilitating its excretion. It is also a tonic—and in children, to a moderate extent, a diuretic—but less so in an adult. It appears to have a peculiar and specific action on all that variety of diseases connected with derangements of the digestive organs, and from the proofs I have advanced, I believe it worthy of extensive trial. The preparation I have been in the habit of giving, is simply the recent gall of the ox slowly evaporated to the consistence of an extract, and afterwards made into pills, as in the formulæ already given; but if it is sufficiently firm, I prefer the simple extract made into pills without any addition; and if the gall be *recent*, it has very little smell, but an intensely bitter taste. The gall-bladder of a moderate sized ox will afford as much extract as will make one hundred four grain pills, and is an article both cheap and easy to procure. Trusting it may be further tested by others, I leave it to the profession, confidently recommending it to their notice.

The value of opium as a remedy in disease is acknowledged by all practitioners, and it would be much more generally given if it were not for the constipation resulting from its application, by the general check it is supposed to give to the operation of the secreting organs. How this is accomplished is not so easily explained; physiologists admitting, that though digestion may progress during sleep, secretion is but very slowly carried on. The action of opium, then, may not be direct on the secretory organs, but by producing artificial sleep, *through that sleep* produce the check to the secretions, generally attributed to the direct effect of the opium itself.

Perspiration, however, may be adduced as a contradiction to this, but it may with equal propriety (as it has been frequently) be denied that perspiration *is a secretion;* this (perhaps properly called) exudation being most certainly and frequently produced by sleep, it also follows that the perspiration attributed to various preparations of opium, "pulv. Doveri, &c." may not be the direct effect of the preparation used, but the consequence of the sleep produced by it. Dr. Holland, in his "Notes and Reflections," observes, "the fear of confining the bowels, and checking the secretions, constantly present to the mind of the practitioner, prevents the adequate use of a medicine, having the power of mitigating pain, relieving spasm, procuring sleep, and producing perspiration, &c."

In whatever view the action of opium is considered, it must be acknowledged that if its action *can* be secured without the disadvantages generally attributed to it, its value as a remedial agent must be very considerably increased, and the frequent objection to its use be done away with. That such *can* be *positively* accomplished by the combination of ox-gall with it, the cases here given fully prove. And supposing the secretion of bile in the system to be deficient in quality, or quantity, originating disease from whatever cause, there cannot remain a doubt but that an artificial supply of bile must be attended with the best possible results; that *substitute* acting in the full capacity of the original secretion, until such secretion be either amended or restored. Ancient pathologists, particularly of the Hippocratic school, attributed considerable importance to bile; and perhaps moderns have too much neglected the results arising from its redundancy or deficiency in the animal system. What I have hitherto advanced has been chiefly to illustrate the deficiency of bile in the alimentary canal; but before I conclude this subject, it may be necessary to observe very briefly, that diseases not unfrequently arise from the bilious secretion being too great in quantity, and what necessarily follows, depreciated in quality; hence those obstinate and long-continued diarrhœas, and cases of jaundice, *accompanied with purging* (*which are very distinct* from the common run of cases of jaundice,) being the consequence of mechanical obstruction in the biliary ducts, always accompanied with constipation, little or no bile passing along the alimentary canal. But I have frequently observed cases of jaundice, and more in infancy than adult ages, where no such obstruction existed, and where motions evidently indicated a full sufficiency of bilious secretion. Jaundice arising from obstruction in the biliary ducts *may*, after the mechanical obstruction has been removed by emetics, &c., be much benefitted by the exhibition of gall internally, which not only assists the bowels in passing off the excrementitious mass, but improves the secretion of bile so as to prevent the formation of gall-stones in future. But those cases accompanied with lax motions, as well as long standing cases of obstinate diarrhœa, would rather be injured than benefitted by it. The only resource in such is to lessen the bilious secretion, and I know of no one object more efficient in checking that secretion, and exciting perspiration, than small and frequent doses of crude opium, with warm clothing. If once the exudation by the skin becomes apparent, in such cases it may very certainly be predicated that the secretion of bile is lessened, and the case progressing towards a cure. I am convinced there exists a remarkable sympathy between the secretion of the liver and the exudation by the skin, either of which being lessened increases the other, and vice versa.

